# *Actinobaculum suis* Detection Using Polymerase Chain Reaction

**DOI:** 10.1100/2012/572732

**Published:** 2012-12-30

**Authors:** Cristina Román Amigo, Debora Dirani Sena de Gobbi, Vasco Túlio de Moura Gomes, Danilo do Prado Perina, Pedro Henrique Nogueira de Lima Filsner, Barbara Letícia Pereira Costa, Maria Garcia Spindola, Thais Sebastiana Porfida Ferreira, Paulo Eduardo Brandão, Andrea Micke Moreno

**Affiliations:** ^1^Departamento de Medicina Veterinária Preventiva e Saúde Animal, Faculdade de Medicina Veterinária e Zootecnia (FMVZ), Universidade de São Paulo (USP), Avenida Professor Dr. Orlando Marques de Paiva 87 Cidade Universitária, 05508 270 São Paulo, SP, Brazil; ^2^Departamento de Zootecnia, Escola Superior de Agricultura “Luiz de Queiroz” (ESALQ), Universidade de São Paulo (USP), Avenida Pádua Dias 11, Agronomia, 13418-900 Piracicaba, SP, Brazil

## Abstract

*Actinobaculum suis* is an important agent related to urinary infection in swine females. Due to its fastidious growth characteristics, the isolation of this anaerobic bacterium is difficult, thus impairing the estimation of its prevalence. The purpose of this study was to develop and test a polymerase chain reaction (PCR) for the detection and identification of *A. suis* and then compare these results with traditional isolation methods. Bacterial isolation and PCR were performed on one hundred and ninety-two urine samples from sows and forty-five preputial swabs from boars. The results indicate that this PCR was specific for *A. suis*, presenting a detection limit between 1.0 × 10^1^ CFU/mL and 1.0 × 10^2^ CFU/mL. *A. suis* frequencies, as measured by PCR, were 8.9% (17/192) in sow urine samples and 82.2% (37/45) in preputial swabs. Assessed using conventional culturing techniques, none of the urine samples were positive for *A. suis*; however, *A. suis* was detected in 31.1% (14/45) of the swabs. This PCR technique was shown to be an efficient method for the detection of *A. suis* in urine and preputial swabs.

## 1. Introduction

*Actinobaculum suis* is a Gram-positive anaerobic rod bacterium involved in serious forms of urinary infection in gilt swine that result in hematuria, cystitis, and pyelonephritis, which can cause animal death. Sow infection occurs through contact with a contaminated environment or through natural mating with carrier boars. A high number of swine males are colonized by *A. suis* in their preputial diverticula, and this colonization begins in the first weeks of life [1]. 

Due to its slow fastidious growth, *A. suis* has been difficult to isolate, a fact which may have impaired estimates of its prevalence. Conventional culturing techniques for the identification of anaerobic bacteria can be time-consuming, are not always economically feasible and are beyond the capabilities of some smaller diagnostic laboratories [2]. As an alternative to direct bacterial isolation, indirect immunofluorescence (IF) has been used for *A. suis *detection [3–7]. However, the disadvantages of the IF technique—such as the need for animals for antibody production, the paucity of antibody production laboratories for this agent, and the requirement of specialized equipment and personnel—make polymerase chain reaction (PCR) an affordable and promising alternative tool for the detection of *A. suis*. Although PCR has not been used as a diagnostic method for this bacterium in pigs, this mechanism is already being applied to detect other species of the genus *Actinobaculum* sp. Bank et al. [8] described a PCR technique for the detection of *Actinobaculum schaalii* in human urine and found PCR to be a rapid and reliable method of detection, which contributed to more effective treatment and faster recovery of patients.

The present study aims to develop a PCR strategy for the detection and identification of *A. suis* in pure cultures, urine samples, and preputial swabs; evaluate the PCR specificity and limits of detection; and compare the PCR results with those obtained using direct bacterial isolation techniques.

## 2. Material and Methods

### 2.1. Sample Collection

One hundred and ninety-two urine samples from sows and forty-five swabs of preputial diverticula from boars were collected from three swine herds in São Paulo State, Southeastern Brazil. Samples were kept at 4°C until processing. 

### 2.2. Bacteriological Examination

Urine samples (10 mL) were centrifuged at 4,000 ×g for 10 minutes, and the obtained pellet or preputial swabs were spread in 5% sheep blood agar supplemented with colistin sulphate (10 mg/L), nalidixic acid (15 mg/L) and metronidazole (50 mg/L). All antimicrobial powders were obtained from Sigma Chemical (St. Louis, MO, USA.). The plates were incubated in anaerobic conditions at 37°C for 72 hours. The colonies presenting a characteristic dry, greyish-white, flattened, opaque surface, without hemolysis, were submitted for biochemical tests and the PCR described below. Morphology, catalase and urease production, hippurate hydrolysis, nitrate reduction, and the fermentation of glucose, starch, lactose, maltose, and trehalose were all tested.

### 2.3. DNA Extraction

Purified DNA was recovered according to the Boom et al. [9] protocol for DNA extraction, following previous enzymatic treatment for 60 min at 37°C with 10 *μ*g of lysozyme (USBiological, Swampscott, MA/USA) and 400 *μ*g of proteinase K (LGC Biotecnologia, Cotia, SP/Brazil), and was then stored at −20°C. 

### 2.4. Primer Design

A pair of specific primers for *A. suis* detection was designed using the 16S ribosomal RNA-coding sequence described by Ludwig et al. [10] (GenBank accession number S83623.1), using Primer BLAST (http://www.ncbi.nlm.nih.gov/tools/primer-blast/index.cgi?LINK_LOC=BlastHome). 

Putative primers suggested by Primer BLAST and those with lower identity with non-*A. suis* sequences were selected, resulting in the pair Acs-1 and Acs-2 (Table 1) (Tms 59.45 and 60.18°C, resp.; positions 86 to 217 of S83623.1).

### 2.5. Polymerase Chain Reaction

Multiple PCR conditions were evaluated, and optimum conditions used for all subsequent tests were: 50 *μ*L reaction containing 1.5 mM of MgCl_2_, 5.0 *μ*L of PCR Buffer, 200 mM dNTP, 20 pmol of each *primer *(Table 1), 1.0 U of *Taq *DNA polymerase (Fermentas Inc., Glen Burnie, Maryland/USA), 5 *μ*L of DNA template, and ultrapure water. PCR was carried out for 35 cycles consisting of denaturation for 1 min at 94°C, annealing for 1 min at 50°C, and extension for 1 min at 72°C. 

The amplified products were detected by means of electrophoresis at 80 V in 1.5% agarose gel stained with Blue Green (LGC Biotecnologia, Cotia, São Paulo/Brazil) for 40 min and were photographed under UV transillumination with the ImageMaster Photo Documentation System (GE Healthcare do Brazil Ltda., São Paulo/Brazil). A 100 bp DNA ladder (New England BioLabs Inc., Ipswich, MA/USA) was used for band size determination.

### 2.6. Detection Limit

 The detection limit of the PCR assay was determined by using a 10-fold serial dilution of known concentrations (1 × 10^1^ to 1 × 10^10^ CFU/mL) of *A. suis* strain LSSU9/11 in phosphate buffered saline (PBS, LGC Biotecnologia, Cotia, São Paulo/Brazil). 

### 2.7. Analytical Specificity

To determine the analytical specificity of the assay, 14 clinical strains of *A. suis* and the LSSU9/11 strain were tested. Phylogenetically related and clinically relevant bacterial strains, including 22 species described in Table 2, were also tested. 

### 2.8. DNA Sequencing

PCR products obtained from three *A. suis* strains (the LSSU9/11 strain and two preputial strains) were gel-purified using the AxyPrep Gel Extraction Kit (Axygen Biosciences, Union City, CA/USA) and were then sequenced with the Acs-1 and Acs-2 (Table 1) primers using BigDye 3.1 (Applied Biosystems, Foster City, CA, USA) and ABI 3500 XL genetic analyzer (Applied Biosystems, Foster City, CA, USA), according to the manufacturer's instructions. Sequences were then submitted to blastn analysis.

### 2.9. Statistical Analysis

The agreement between the PCR and isolation results was estimated using the Kappa test [11].

## 3. Results

PCR using primers specifically designed for *A. suis* showed a detection limit between 1 × 10^1^ CFU/mL and 1 × 10^2^ CFU/mL and generated a 133 bp band. None of the twenty-three strains from different species tested were positive according to PCR, whereas all fourteen *A. suis* strains isolated from urine by culture technique were positive. Sequencing of amplicons obtained from three *A. suis* strains showed a 98% similarity with the sequence of *A. suis* 16S (NR 044760.1) deposited in GenBank and only a 90% similarity when compared with three *Actinobaculum massiliense* isolates.

Among the 237 samples processed, PCR detected 22.8% (54/237) of positives for *A. suis,* while traditional culturing indicated only 5.9% (14/237) of positives (Table 3). From the urine samples, PCR detected *A. suis* in 8.9% (17/192) of the samples, and the isolation procedure did not identify any positive samples. From the preputial swabs, *A. suis* was detected by PCR in 82.2% (37/45) of the samples, and isolated in 31.1% (14/45). The analysis of agreement between techniques encompassing all samples showed a Kappa value of 0.358, which is considered a weak level of agreement. 

## 4. Discussion

Considering the difficulties involved in isolating bacteria due to the growth features of *A. suis *in veterinary diagnostic laboratories—the need for antimicrobial supplemented media, time-consuming incubation (72 hours), and laborious biochemical tests—the importance and prevalence of this agent in swine herds in Brazil and worldwide are often underestimated, because of the low-sensitivity detection methods currently in use. 

To date, there have been no reports on the use of molecular tests for the detection of *A. suis *in swine. Bank et al. [8] described a PCR protocol for the detection of *Actinobaculum shaalii* in human urine samples, using specific primers for the gyrase B (*gyr*B) gene. For the present this study, the primers were designed using the 16S ribosomal RNA sequence deposited in GenBank and described by Ludwig et al. [10], which is a highly conserved gene in bacterial genera, widely used in targeted detection and typing. 

The detection limit of the PCR assay described herein was between 10 and 100 CFU/mL, which is compatible with several publications concerning molecular diagnostic tools but inferior to that described by Bank et al. [8], who reported 1.5 × 10^3^ to 1.5 × 10^4^ CFU/mL of *Actinobaculum shaalii *in urine samples using real-time PCR. The evaluation of the analytical specificity using different swine pathogens, including *Arcanobacterium pyogenes*, the phylogenetically closest bacterium to the *Actinobaculum* genus [12], showed no reaction. Sequences from amplicons obtained with these new primers matched *A. suis* sequences. Furthermore, when primers were tested in clinical samples, nonspecific bands were not observed. 

Because the aim of the present study was not to evaluate the prevalence of *A. suis *in urine or preputial swabs, but rather to compare the two methodologies tested, a statistically significant sampling was not carried out. Nonetheless, the comparison of the results obtained herein with other *A. suis* frequency reports is key to determining the potential use of PCR in future research involving this bacterium.

Considering the presence of *A. suis* in the urine samples analyzed, it was found that the isolation method did not detect any samples positive for *A. suis* among the 192 samples processed, while PCR detected 8.9%. Reis et al. [13], and Menin et al. [14] isolated the agent from 2.0% (1/60) and 4.0% (37/922) of the cases examined in Brazil. Vaz et al. [6] and Porto et al. [4] reported *A. suis* prevalence rates of 16.8% (17/101) and 31.4% (11/35), respectively, using IF in Brazilian herds. The frequency found in the present study lies within the ranges previously described.

From the preputial swab samples, isolation demonstrated 31.1% (14/45) positivity for *A. suis,* while PCR detected 82.2% positivity (37/45). These values are in accordance with prevalence studies previously conducted in Brazil, which reported rates of 53.8% (21/39) by means of isolation and 78.0% (75/96) by means of IF [5, 15].

Other groups have previously reported the occurrence of *A. suis* in boar preputial swabs around the world. These include Pijoan [16], with a 60.5% (23/38) positivity rate in the United States samples, and Jones and Dagnall [17], who indicated 89.0% (200/224) positivity for *A. suis* in the United Kingdom samples, both using isolation procedures. Sobestiansky et al. [5] reported 67.0% (52/78) positive swabs in Portugal, and 76.0% (16/21) positive swabs in Argentina by means of IF.

In this study, a comparison between the PCR technique developed here and the isolation and IF techniques was not possible. However, the results indicate a higher efficacy for the PCR method compared to isolation, as the number of positives detected by PCR in preputial swabs and urine were comparable to the rates observed using IF. Presently, use of molecular tools is widespread in research and veterinary diagnostic laboratories and is considered accessible and affordable.

The Kappa value of 0.358 indicates a very weak concordance between PCR and isolation methods, indicating that PCR is a very efficient tool for epidemiological and diagnostic studies of *A. suis* infections in swine herds when compared with the traditional isolation method. This is partly due to the ability of PCR to detect specific genome fragments from viable as well as dead bacteria. This result is even more important when considering this agent's previously described growth traits and the absence of nonspecific PCR amplicons for all samples tested. 

In conclusion, the PCR developed and tested in this study is a fast and reliable tool for *A. suis* detection, even when the agent is present in small quantities together with other bacteria in its original environment, such as the urinary tract or preputial diverticula.

## Figures and Tables

**Table 1:** Primer design for *A. suis* PCR detection.

Primer	Sequence (5′-3′)	Amplicon size (bp)
Acs1	CGTGGGTAACCTGCCCTCAACTG	133
Acs2	CAAACTGATAGGCCGCGAGCCC	

**Table 2:** Bacteria species used to test analytical specificity of PCR for *Actinobaculum suis*.

Species	Source/identification
*Actinobaculum suis *	Clinical isolates (14)
*Actinobacillus pleuropneumoniae *	ATCC 27089
*Arcanobacterium pyogenes *	ATCC 19411
*Arcobacter butzleri *	ATCC 49616
*Arcobacter cryaerophilus *	ATCC 43158
*Bordetella bronchiceptica *	ATCC 4617
*Brachyspira hyodysenteriae *	ATCC 27164
*Brachyspira pilosicoli *	ATCC 51139
*Campylobacter coli *	ATCC 43478
*Campylobacter jejuni *	ATCC 33292
*Clostridium perfringens *	ATCC 12922
*Erysipelothrix rhusiopathiae *	ATCC 19414
*Escherichia coli *	ATCC 11105
*Haemophilus parasuis *	Clinical isolate
*Klebsiella pneumoniae *	ATCC 10031
*Listeria monocytogenes *	ATCC 7644
*Mycoplasma hyopneumoniae *	ATCC 25095
*Mycoplasma hyorhinis *	ATCC 17981
*Pasteurella multocida *	ATCC 43137
*Salmonella typhimurium *	ATCC 14028
*Staphylococcus aureus *	ATCC 25923
*Staphylococcus hycus *	Clinical isolates
*Streptococcus suis *	Clinical isolates

**Table 3:** Results of PCR and isolation of *A. suis* from urine and preputial swabs.

Sample	PCR	Isolation	
		Positive	Negative
Urine	Positive	0	17 (8.9%)
	Negative	0	175 (91.1%)

Preputial swab	Positive	14 (31.1%)	23 (51.1%)
	Negative	0	8 (17.8%)
